# The Effect of Modified Sini Decoction on Survival Rates of Patients with Hepatitis B Virus Related Acute-on-Chronic Liver Failure

**DOI:** 10.1155/2019/2501847

**Published:** 2019-02-24

**Authors:** Jian-Xing Luo, Yang Zhang, Xiao-Yu Hu, Chang Yu

**Affiliations:** ^1^Department of Infectious Diseases, Hospital of Chengdu University of Traditional Chinese Medicine, Chengdu 610072, Sichuan, China; ^2^Department of Clinical Medicine, Chengdu University of Traditional Chinese Medicine, Chengdu 610075, Sichuan, China; ^3^Acupuncture and Tuina College, Chengdu University of Traditional Chinese Medicine, Chengdu 610075, Sichuan, China

## Abstract

*Aim of the Study. *To verify the effect of modified sini decoction on patients with hepatitis B virus related acute-on-chronic liver failure.* Materials and Methods. *A retrospective cohort study was conducted. Patients who had been treated with modified sini decoction and standard comprehensive internal medicine were assigned to an observation group, and patients who had been treated with standard comprehensive internal medicine were selected as a control group. The total bilirubin (TBIL), albumin (ALB), alanine aminotransferase (ALT), prothrombin activity (PTA), CTP, and MELD scores were analyzed at weeks 4, 8, and 12 after treatment, respectively. Meanwhile, the 12-week survival rate was analyzed.* Results. *The levels of TBIL and ALT were remarkably decreased, while the levels of ALB and PTA were remarkably increased in both groups at weeks 4, 8, and 12 after treatment, respectively, but the effects in the observation group were greater (P < 0.05). The CTP and MELD scores at 8-week and 12-week were lower in the observation group than in the control group (P < 0.05). At 12 weeks, the mean survival times of the observation group and the control group were 66.7 and 45.5 d, respectively. Significant improvement of 12-week survival rate [39/62 (62.9%) versus 18/50 (36.0%), P = 0.001] was observed in the observation group after treatment.* Conclusions. *Modified sini decoction could protect the liver function and improve the survival rates of patients with hepatitis B virus related acute-on-chronic liver failure.

## 1. Introduction 

Liver failure has various clinical types, a complex pathogenesis, and rapid disease progression, as well as a high mortality rate [1]. Acute-on-chronic liver failure (ACLF) is one type of it. ACLF is characterized by an acute deterioration with preexisting chronic liver diseases and ultimately results in increased mortality due to multisystem organ failure [2]. In most Asian countries, the aetiology of ACLF is primarily due to hepatitis B virus (HBV) infection because of the high prevalence of it [3]. As we all know, HBV has a worldwide distribution and is endemic in many populations [4]. 90% of ACLF is caused by chronic HBV infection in China [5]. So hepatitis B virus related acute-on-chronic liver failure (HBV-ACLF) caused by HBV infection is the most common type in China with various complications and a mortality rate as high as 40%-90% [1, 6, 7]. At present, liver transplantation is the most effective treatment, which can increase the survival rates of patients with liver failure [5]. But it is severely limited by high costs and donor shortage [8, 9]. Therefore, it is time to explore a new strategy to cure HBV-ACLF.

Previous study has reported the effects of traditional Chinese medicine (TCM) on HBV-ACLF, including improvement of symptoms, amelioration of liver function, and reduction of the risk of adverse reactions [10]. So we can continue to identify a novel strategy to cure HBV-ACLF from TCM. Sini decoction is a well-known traditional Chinese herbal formulation, which has been used to treat cardiovascular diseases and liver diseases [11–13] since the Han Dynasty, 200–210A.D. According to traditional Chinese medical theory, we created the formula “modified sini decoction” (MSND) to treat liver failure in the Hospital of Chengdu University of Traditional Chinese Medicine [14]. In our previous study [14], MSND was used to treat acute liver failure induced by D-galactosamine in rats. It was useful to protect the liver function and improve the survival rates of acute liver failure rats. So in order to investigate the effect of MSND on patients with hepatitis B virus related acute-on-chronic liver failure, we conducted this clinical trial.

## 2. Materials and Methods

### 2.1. Study Design

The retrospective cohort study was conducted at the Department of Infectious Diseases, Hospital of Chengdu University of Traditional Chinese Medicine from October 2011 to June 2015. Patients with HBV-ACLF formed an observation cohort or a control cohort according to whether they took TCM therapy or not. Patients in the observation cohort were given MSND and standard comprehensive internal medicine, while patients in the control cohort were only given standard comprehensive internal medicine. There were no artificial liver support system and liver transplantation. All patients or their immediate family signed the informed consent. According to the previous studies [10, 15], the survival rate in the control group was set to be 35%, while the survival rate in the observation was set to be 60% in this study. The match ratio, type I error, and power of test were 1:1, 5%, 80%, respectively [10, 16]. The sample size was 50 in each group, and the target sample size was 112 with an expulsion rate of 10%.

### 2.2. Inclusion Criteria

The inclusion criteria were [17, 18] (1) serum total bilirubin ≥ 171 *μ*mol/L or a daily increase ≥ 17.1 *μ*mol/L, (2) international normalized ratio (INR) ≥ 2.6 or prothrombin activity ≤ 20% without other reasons, (3) with hepatic encephalopathy or severe infection or hemorrhage of upper gastrointestinal tract or hepatorenal syndrome, (4) the presence of hepatitis B surface antigen in the serum for at least 6 mo, (5) age from 18 to 65 years, and (6) patients with Yang Deficiency of Spleen and Kidney who met diagnosis standards of Chinese medicine syndrome differentiation [19].

### 2.3. Exclusion Criteria

The exclusion criteria were (1) age < 18 years or age > 65 years; (2) patients with acute liver failure, subacute liver failure, or chronic liver failure; (3) superinfection or coinfection with hepatitis A, C, D, E viruses, or human immunodeficiency virus; (4) coexistence of any other serious systemic or psychiatric diseases; (5) coexistence of any other liver diseases, such as alcoholic liver disease, Wilson's disease, drug hepatitis, hepatocellular carcinoma, or autoimmune hepatitis; (6) women during pregnant stage and breast-feed stage; (7) prolonged prothrombin time induced by blood system disease; and (8) patients who had taken any illicit drugs or Chinese herbal products for any disease within the preceding 12 mo.

### 2.4. Treatments

All patients were given standard comprehensive internal medicine [20] in this study. Patients who accepted standard comprehensive internal medicine alone were selected as a control group. Patients who were treated by standard comprehensive internal medicine and TCM therapy (modified sini decoction) were considered an observation group. The composition of modified sini decoction consisted of* Aconitum carmichaelii*,* Zingiber officinale*,* Glycyrrhiza uralensis*,* Panax ginseng*,* Prunus mume*, etc. TCM clinicians tailored the formula based on changes in the syndrome and the patients' conditions.

### 2.5. Clinical and Laboratory Data

The patients' clinical and laboratory data were collected: (1) the 12-week survival rate; (2) the Child-Turcotte Pugh (CTP) score [21] and model for endstage liver disease (MELD) score [22] before and after treatment for 4, 8, and 12 weeks; (3) biochemical indicator: serum ALT, AST, total bilirubin (TBIL), and albumin (ALB) at 0, 4, 8, and 12 weeks after treatment, respectively; all assays used a colorimetric method (Automatic Analyzer 7170A, Hitachi, Japan); (4) PTA was performed following the manufacturer's instructions (STA-evolution, STAGO, France). It was tested at 0, 4, 8, and 12 weeks after treatment, respectively.

### 2.6. Safety Assessment

In this study, all patients were questioned about adverse events. Every adverse event including feelings of discomfort or unexpected symptoms was recorded. The start date, end date, and degree of each event were recorded. Patients with any adverse event received appropriate treatment.

### 2.7. Statistical Analysis

Patients' survival data were evaluated by the Kaplan–Meier method and compared by the log-rank test. Quantitative data were expressed as the means ± standard deviation. Differences between quantitative data were compared by independent-samples t-test or Mann–Whitney U tests. Differences between qualitative data were analyzed by *χ*2 test or Fisher's exact test. Statistical testing was performed with the SPSS software for Windows, version 17.0 (SPSS Inc., USA). All P values were two-sided, and P < 0.05 was considered to be statistically significant.

## 3. Results 

### 3.1. Patients

**Figure 1:**
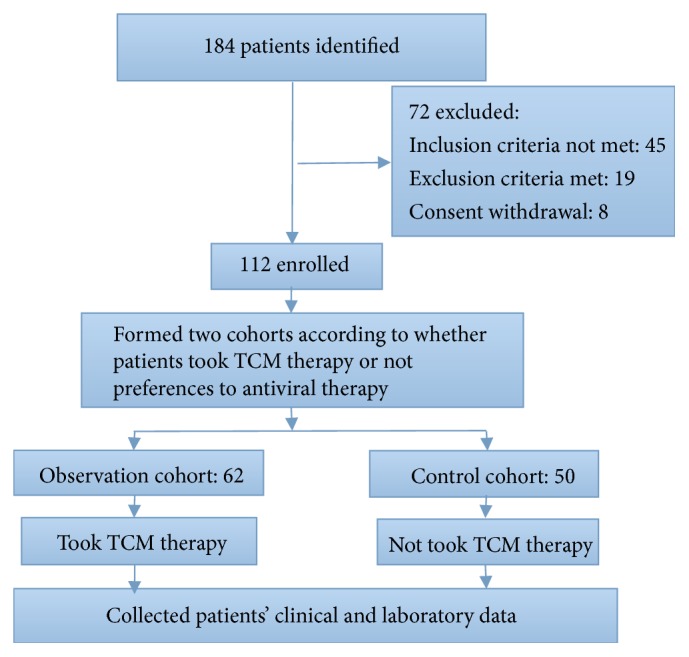
The research flowchart.

One hundred and twelve patients were enrolled from 184 cases with hepatitis B virus related acute-on-chronic liver failure from Hospital of Chengdu University of Traditional Chinese Medicine. There were 62 patients in the observation group and 50 patients in the control group. The flowchart is shown in Figure 1.

### 3.2. Baseline Characteristics

No significant differences were found in gender, age, serum ALT, AST, TBIL, ALB, PTA, HBV-DNA levels, HBeAg (±), CTP, and MELD scores (p > 0.05). And there were no differences in complications between the two groups before treatment (p > 0.05). The baseline characteristics of patients are shown in Table 1.

### 3.3. 12-Week Survival Rate

**Figure 2:**
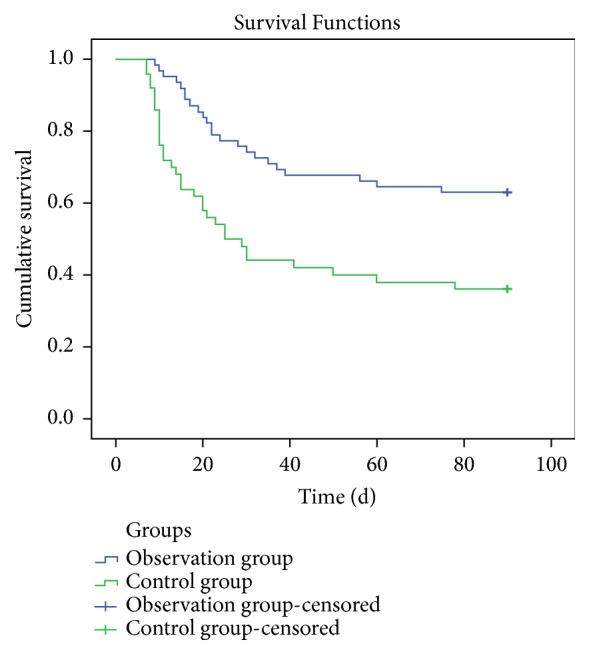
Survival curve of patients in the two groups after 12-week treatment.

There were 39 (62.9%) cases survived in the observation group and 18 (36.0%) patients survived in the control group after 12-week treatment. The mean survival time of the patients in the observation group was 66.7 d, while the mean survival time was 45.5 d in the control group. Compared to the control group, the cumulative survival rate was significantly improved in the observation group (P = 0.001). The results are shown in Figure 2.

### 3.4. Biochemical Characteristics

As for the ALT and AST levels between the two groups, there were statistical differences at week 4 (P < 0.05), but no significant difference at weeks 8 and 12 (all P > 0.05). As time went on, the TBIL levels were decreased in both groups, while the ALB and PTA were increased in both groups. But the effects were greater in the observation group at 4, 8, and 12 weeks, respectively (P<0.05). The results are shown in Tables 2–4.

### 3.5. CTP and MELD Scores

The CTP and MELD scores in both groups were decreased after treatment. But the CTP and MELD scores at 8-week and 12-week were lower in the observation than in the control group (P < 0.05). The results are shown in Tables 2–4.

### 3.6. Safety

No patient developed severe drug-induced adverse events.

## 4. Discussion 

Chronic liver disease is a serious health problem. Among it, chronic HBV infection is the most difficult one worldwide [5]. China has always maintained a high morbidity of chronic HBV infection. And in China, approximately 90% of ACLF is induced by chronic HBV infection [23]. HBV-ACLF is a devastating syndrome with extremely high mortality [24]. Multiple organ failure is the main cause of death in 77% of patients with HBV-ACLF [5]. As all we know, the prognosis of HBV-ACLF depends on the capability of liver cell regeneration, the degree of hepatic damage, and the prevention of multisystem organ failure. Liver transplantation [16] has been the only definitive therapy to salvage these patients; however, it is not readily available and feasible in many parts of the world, and the artificial liver support system cannot fully meet the needs [16]. Therefore, it is time to identify a novel strategy to cure HBV-ACLF.

In China, traditional Chinese medicine (TCM) has been used to cure diseases successfully for centuries [25, 26]. In our previous study [14], we used MSND to treat acute liver failure in rat induced by D-galactosamine. Our results indicated that MSND could improve liver function, reduce necrosis in the liver tissues, and remarkably prolong the survival time. It meant that MSND could gain more time for the regeneration of liver cells. So MSND might be a useful means to save these patients. And in this retrospective cohort, our results indicated that MSND could reduce CTP and MELD scores at weeks 8 and week 12 in patients with HBV-ACLF (P<0.05). Meanwhile, MSND could improve the biochemical parameters of patients with HBV-ACLF. The results were mainly reflected by the decreasing of ALT and AST at week 4 (P<0.05), the decreasing of IBIL at 4, 8, and 12 weeks, respectively (P<0.05), and the increasing of ALB at 4, 8, and 12 weeks, respectively (P<0.05). But there were no significant differences (P>0.05) at week 8 and week 12 for ALT and AST levels between the two groups. The possible reason might be that after massive necrosis of hepatocytes in patients with hepatitis B virus related acute-on-chronic liver failure, transaminase has maintained a high level for quite a long time, and transaminase would be progressively exhausted; finally, ALT and AST would decrease or even be normal. So there were no significant differences at week 8 and week 12. At the same time, MSND could significantly increase PTA at 4, 8, 12 weeks, respectively (P<0.05). Thus, MSND could prolong the survival times of the hepatitis B virus related acute-on-chronic liver failure patients finally. Therefore, it could be concluded that MSND was a useful therapeutic agent for HBV-ACLF.

In our study, the formula MSND consists of* Aconitum carmichaelii*,* Zingiber officinale*,* Glycyrrhiza uralensis*,* Panax ginseng, Prunus mume*, etc. The formula has warming and nourishing yang-qi effect in deficiency of vital energy and eliminating cold-dampness. Previous studies [13, 27–32] showed that the Chinese medicines used in this study had widespread biological activities, including protecting the damaged hepatocytes. And our previous [14] study had explored the possible mechanisms of the effects of MSND on protecting liver function. Our results revealed that MSND could decrease the expressions of the HMGB1, TLR4, and NF-*κ*B mRNAs, downregulate the expression of Caspase-3 mRNA to reduce apoptosis in hepatocytes, and increase the PCNA-positive rate [14].

In addition, the trial had limitations. A retrospective study is conducted by looking backwards at clinical events that have occurred. The trial cannot follow the randomization principle. The selection bias is the main disadvantage of the retrospective study. The randomized, controlled trials (RCT) have the highest grade evidence, but HBV-ACLF is such a serious disease, it was not ethical to conduct an RCT. And a prospective study needs larger size and takes too long to be conducted. Therefore, a retrospective cohort study was conducted by us. At the same time, in order to improve the validity of this study, we set an inclusion and exclusion criteria, and so on.

## 5. Conclusion

This trial showed that modified sini decoction could protect the liver function and improve the survival rates of patients with hepatitis B virus related acute-on-chronic liver failure. This study might provide an effective and safe option for patients with HBV-ACLF.

## Figures and Tables

**Table 1:** Baseline characteristics.

Characteristics	Observation group	Control group	t/*χ*^2^	*P*-value
Age (years)	41.7 ± 9.9	42.5 ± 10.2	1.341	0.381
Males (%)	43(69.4)	35(70.0)	0.005	0.941
ALT (U/L)	438.5 ± 85.8	456.9 ± 89.6	0.594	0.618
AST (U/L)	401.7 ± 78.5	410.7 ± 86.3	0.641	0.431
TBIL (umol /L)	356.3 ± 80.5	347.6 ± 78.1	0.684	0.513
PTA (%)	17.1 ± 2.4	16.7 ±3.2	0.298	0.701
ALB (g/L)	26.1 ± 6.4	26.7 ±6.0	0.562	0.487
Creatinine (umol/L)	72.3 ± 15.1	69.6 ± 12.9	1.270	0.301
Sodium (mmol/L)	130.9 ± 38.3	129.1 ± 40.5	1.615	0.231
CTP points	12.2 ± 1.7	13.0 ± 2.3	1.304	0.209
MELD points	28.9 ± 5.1	26.7 ± 4.9	0.732	0.809
HBeAg-positive (%)	21 (33.9)	18 (36.0)	0.055	0.814
HBV-DNA (log10 copies/mL)	6.4 ± 1.3	6.7 ± 1.8	0.781	0.512
Ascites (%)	50 (80.6)	37 (74.0)	0.705	0.401
Hepatic encephalopathy(II-IV) (%)	22 (35.5)	17 (34.0)	0.027	0.870
Severe infection (%)	16 (25.8)	13 (26.0)	0.001	0.981
Hemorrhage of upper gastrointestinal tract (%)	21 (33.9)	18 (36)	0.055	0.814
Hepatorenal syndrome (%)	6 (9.7)	5 (10.0)	0.003	0.955

All values are expressed as mean ± SD or number (%).

**Table 2:** Baseline characteristics at 4 weeks.

Characteristics	Observation group	Control group	t	*P*-value
ALT (U/L)	214.2 ± 71.7	273.2 ± 80.5	2.936	0.018^★^
AST (U/L)	215.9 ± 77.2	269.4 ± 76.2	2.538	0.040^★^
TBIL (umol /L)	261.7 ± 62.3	301.9 ± 72.5	3.732	0.001^★^
PTA (%)	34.3 ± 8.3	27.9 ±9.8	2.151	0.032^★^
ALB (g/L)	29.5 ± 6.8	27.0 ±6.5	2.653	0.048^★^
CTP points	8.9 ± 2.1	10.1 ± 2.8	0.988	0.056
MELD points	22.5 ± 5.9	24.1 ± 4.9	0.547	0.094

All values are expressed as mean ± SD. ^★^Statistical significant difference between groups.

**Table 3:** Baseline characteristics at 8 weeks.

Characteristics	Observation group	Control group	t	*P*-value
ALT (U/L)	56.7 ± 14.6	60.4 ± 16.4	0.849	0.115
AST (U/L)	70.5 ± 20.8	74.3 ± 22.9	0.641	0.189
TBIL (umol /L)	127.3 ± 39.8	196.3 ± 70.1	2.942	0.001^★^
PTA (%)	50.2 ± 10.8	38.3 ±9.6	1.843	0.015^★^
ALB (g/L)	33.6 ± 7.9	28.6 ±6.7	2.093	0.032^★^
CTP points	6.8 ± 2.3	8.9 ± 3.2	1.085	0.037^★^
MELD points	14.6 ± 3.7	18.5 ± 4.1	1.867	0.026^★^

All values are expressed as mean ± SD. ^★^Statistical significant difference between groups.

**Table 4:** Baseline characteristics at 12 weeks.

Characteristics	Observation group	Control group	t	*P*-value
ALT (U/L)	47.3 ± 13.8	51.1 ± 14.8	0.913	0.203
AST (U/L)	51.4 ± 15.6	57.8 ± 16.7	0.863	0.392
TBIL (umol /L)	58.5 ± 25.3	102.5 ± 39.6	2.649	0.001^★^
PTA (%)	62.5 ± 11.7	46.7 ±10.9	2.537	0.012^★^
ALB (g/L)	38.5 ± 8.2	29.9 ±6.8	2.363	0.021^★^
CTP points	5.3 ± 2.5	8.2 ± 4.1	2.043	0.025^★^
MELD points	10.5 ± 3.2	14.7 ± 4.5	1.992	0.032^★^

All values are expressed as mean ± SD. ^★^Statistical significant difference between groups.

## Data Availability

The data used to support the findings of this study are included within the article.

## Abbreviations

ALT: Alanine aminotransferase
AST: Aspartate aminotransferase
TBIL: Total bilirubin
PTA: Prothrombin activity
ALB: Albumin
CTP: Child-Turcotte Pugh
MELD: Model for endstage liver disease
ACLF: Acute-on-chronic liver failure
HBV: Hepatitis B virus
TCM: Traditional Chinese medicine.
